# Synergistic Effect of H_2_O_2_ and NO_2_ in Cell Death Induced by Cold Atmospheric He Plasma

**DOI:** 10.1038/srep29098

**Published:** 2016-07-01

**Authors:** Pierre-Marie Girard, Atousa Arbabian, Michel Fleury, Gérard Bauville, Vincent Puech, Marie Dutreix, João Santos Sousa

**Affiliations:** 1 Institut Curie, PSL Research University, CNRS UMR3347, INSERM U1021, 91405, Orsay, France; 2 Université Paris-Sud, Université Paris-Saclay, rue Georges Clémenceau, 91405 Orsay, France; 3 LPGP, CNRS, Université Paris-Sud, Université Paris-Saclay, 91405 Orsay, France

## Abstract

Cold atmospheric pressure plasmas (CAPPs) have emerged over the last decade as a new promising therapy to fight cancer. CAPPs’ antitumor activity is primarily due to the delivery of reactive oxygen and nitrogen species (RONS), but the precise determination of the constituents linked to this anticancer process remains to be done. In the present study, using a micro-plasma jet produced in helium (He), we demonstrate that the concentration of H_2_O_2_, NO_2_^−^ and NO_3_^−^ can fully account for the majority of RONS produced in plasma-activated buffer. The role of these species on the viability of normal and tumour cell lines was investigated. Although the degree of sensitivity to H_2_O_2_ is cell-type dependent, we show that H_2_O_2_ alone cannot account for the toxicity of He plasma. Indeed, NO_2_^−^, but not NO_3_^−^, acts in synergy with H_2_O_2_ to enhance cell death in normal and tumour cell lines to a level similar to that observed after plasma treatment. Our findings suggest that the efficiency of plasma treatment strongly depends on the combination of H_2_O_2_ and NO_2_^−^ in determined concentrations. We also show that the interaction of the He plasma jet with the ambient air is required to generate NO_2_^−^ and NO_3_^−^ in solution.

Cancer is a leading cause of death worldwide and its incidence rate increases with the age of the population, the exposure to carcinogens and the modern lifestyle of the population. About two thirds of patients defeat their disease, and the combined action of surgery, radiotherapy and chemotherapy accounts for most cured cases^1^. Alongside with these classical therapies, new therapies have emerged, such as anti-angiogenic therapy and immunotherapy^1^. However, therapy resistance has been observed with every type of therapy that is available today, including poly-chemotherapy, radiotherapy, immunotherapy, and molecular targeted therapy^2^. Importantly, sequencing of primary tumors has revealed that therapy-resistant clones already exist prior to targeted therapy, demonstrating that tumor heterogeneity in primary tumors confers a mechanism for inherent therapy resistance^2^. Therefore, there is still the need of a new therapy that can overcome this problem.

There are numerous publications showing that cold atmospheric pressure plasmas (CAPPs) are effective against tumour cells both *in vitro* and *in vivo* (ref. 3 and references therein). CAPPs are partially ionised gases containing a complex and reactive environment consisting of ions, electrons, free radicals, strong localised electric field, UV radiation, and neutral molecules. CAPPs’ devices are classified in three categories: direct plasma sources that use the target as a counter electrode [*e.g*. floating electrode dielectric barrier discharge (FE-DBD)]; indirect plasma sources that do not use the target as a counter electrode (e.g. plasma jets); and hybrid plasma sources that combine the benefits of direct and indirect plasma sources^4^,^5^,^6^,^7^,^8^,^9^,^10^. Different gases can be used to produce CAPPs such as Helium (He), Argon (Ar), Nitrogen (N_2_), ambient air, or a mixture of gases^6^,^7^. All the plasma sources developed for biomedical applications have in common that the major reactive molecules produced in CAPPs emerge when the components of the partially ionized gas (atoms, molecules, ions and electrons) interact with the molecules of the surrounding air, *i.e*. O_2_, N_2_ and H_2_O, and with the biological sample which is usually a wet surface (*e.g*. cells in medium)^11^,^12^,^13^,^14^. Consequently, the plasma composition and the subsequent effects on cells can vary enormously depending on the plasma source, the plasma settings, the ambient conditions and the biological target^12^,^15^.

Despite this large variability in the plasma composition, it is now widely accepted that the principal mode of plasma-cell interaction is the delivery of reactive oxygen species (ROS) and reactive nitrogen species (RNS) that can be generated in or transferred into the liquid phase surrounding the biological target^16^,^17^. Both short-lived (O, ^•^OH, O_2_^•−^, ^1^O_2_, NO^•^, NO_2_^•^) and long-lived (H_2_O_2_, NO_2_^−^, NO_3_^−^, O_3_) species have been detected in the CAPPs but also in the plasma-treated liquids^17^. However, several groups have shown that the anti-cancer activity of CAPPs was as effective *in vitro* whether the cells, in cell culture medium or in buffer solution, were directly exposed to plasma treatment, or the cell culture medium or buffer solution was first exposed to plasma treatment (so-called plasma-activated medium or plasma-stimulated medium or conditioned medium) and then added to medium-free cells^14^,^18^,^19^,^20^,^21^,^22^,^23^,^24^. This implies that long-lived species play a major role in *in vitro* anti-cancer capacity of CAPPs. Indeed, several publications have shown that H_2_O_2_ (hydrogen peroxide), NO_2_^−^ (nitrite) and NO_3_^−^ (nitrate) are formed at concentrations ranging from μM to mM in CAPP-treated solutions^12^,^14^,^22^,^24^,^25^,^26^, H_2_O_2_ being a central player in the cytotoxicity of CAPPs^21^,^27^,^28^,^29^. The aim of this study was to identify the main long-lived reactive species generated in a simple buffered solution by a He plasma jet operating in ambient air at low gas flow, and their contribution to the plasma-induced cell death in normal and cancer cell lines.

## Materials and Methods

### Cell culture

Normal human skin fibroblasts (NHSF) were kindly provided by Dr Meng-Er Huang (Institut Curie, Orsay, France) and were used at passages below 12. MRC5Vi is a SV40-transformed and immortalized cell line derived from the normal human lung fibroblasts MRC5^30^. HCT116 are human colon cancer cells and Lu1205 are human melanoma cell lines. Dulbecco’s modified Eagle Medium (DMEM) with 4.5 g/l glucose, L-glutamine (L-gln) 100X, penicillin-streptomycin 100 × (10000 U/ml) and fetal calf serum (FCS) were from Eurobio (France). The cells were grown in DMEM containing 10% FCS, P/S 1X and L-gln 1X at 37 °C, 5% CO_2_ in an humidified atmosphere. The cells were regularly checked for mycoplasma contamination using Venor^®^GeM Advance Mycoplasma Detection Kit (Biovalley, France).

### Specifications of the experimental plasma systems

**Figure 1:**
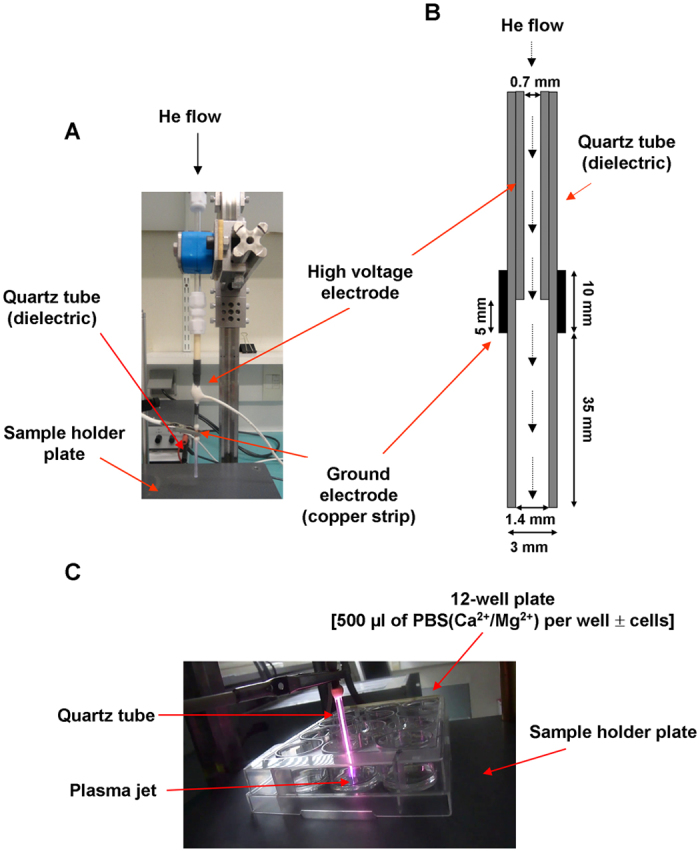
Scheme of the plasma device. (**A**) Photograph of the home-made plasma jet system. (**B**) Schematic illustration of the plasma jet used in this study. (**C**) Photograph showing the interaction between the plasma jet and a solution of PBS(Ca^2+^/Mg^2+^) poured in a 12 well plate.

The plasma source used in this study is a nanosecond pulsed atmospheric pressure cold plasma micro-jet. It consists of a stainless steel needle, inserted inside a dielectric tube made of quartz. The needle is connected to a homemade high voltage generator, while the ground electrode, made of copper, is wrapped around the dielectric tube. Refer to Fig. 1A,B for more details regarding the relative position of the different constituents and their dimensions. The plasma is created by a dielectric barrier discharge (DBD) with axial symmetry by applying high voltage pulses (amplitude of 8 kV, rise time of 280 ns and full width at half maximum of 540 ns) at a repetition rate of 20 kHz to the internal electrode^31^. Pure helium (Alphagaz 2 He type S11, Air Liquide, France) is injected through the needle at a flow rate of 50 or 400 sccm (cm^3^/min), regulated by a flowmeter (GF40-SA46, Brooks instrument, Serv’Instrumentation, France). In these experimental conditions, the plasma jet propagates for about a 1 cm through ambient air outside the quartz tube. The micro-plasma jet was set up vertically with the gas flowing downwards for interaction with buffered solutions covering the various cellular models adhered to the bottom of plate wells. The plasma propagated through a capillary tube, and either the plasma or its gaseous effluent entered the buffer solutions with little admixture of the surrounding air (Fig. 1C).

Another plasma reactor allowing the shielding of the plasma jet with a gas of pure O_2_ (Air Liquide, France), instead of ambient air, was used to evaluate the contribution of the gaseous environment to the toxicity of the He plasma jet. The plasma jet structure, albeit different, is very similar to the other one. Refer to Figure S1 for more details regarding the relative position of the different constituents and their dimensions. The oxygen flow was set to 5 slm and the He flow to 100 or 400 sccm. The plasma is created by applying high voltage pulses (amplitude of 5.5 kV, rise time of 110 ns and full width at half maximum of 260 ns) at a repetition rate of 20 kHz to the internal electrode.

Given the dimensions of both plasma sources and the flows used, Reynolds numbers (Re) between 7 and 55 can be determined. The flows used in this study were, thus, laminar, with very similar Re for both plasma sources (e.g. for 400 sccm, Re = 55 vs Re = 48 in the first (cf. Fig. 1) and second (cf. Figure S1) plasma setup, respectively).

### Plasma treatment

In *in vitro* experiments, 1 × 10^5^ to 4 × 10^5^ cells (depending on the cell type) were seeded per well in 12-well plates and incubated for 24 to 72 h so that the cells are between 50 to 70% confluent at the time of plasma treatment. For direct plasma treatment, cell culture medium was removed, the cells washed 2 times with phosphate buffered saline containing 0.9 mM CaCl_2_ and 0.49 mM MgCl_2_ [called PBS(Ca^2+^/Mg^2+^) in this manuscript], and 500 μl of PBS(Ca^2+^/Mg^2+^) were added to the cells. The cells were then exposed to He plasma in open air for different times, as shown in Fig. 1C. At the end of the plasma treatment, the plates were left at room temperature for 1 h protected from light. For indirect plasma treatment, 500 μl of PBS(Ca^2+^/Mg^2+^) were added to each well of a 12-well plate and treated with He plasma, resulting in plasma-activated PBS(Ca^2+^/Mg^2+^). Parallel to that, the cell culture medium was removed from wells where cells had been incubated, the cells washed 2 times with PBS(Ca^2+^/Mg^2+^) and then exposed for 1 h to the plasma-activated PBS(Ca^2+^/Mg^2+^). In both cases (direct and indirect treatment), 2.5 ml of DMEM containing 10% FCS, P/S 1X and L-gln 1X were added to the cells afterwards, and the plates were incubated at 37 °C and 5% CO_2_ in a humidified atmosphere for 24 h. No difference in cell behaviour between cultures exposed to the gas flow and unexposed cultures has been observed. For shielding experiments, wells of 12 well plates were filled with 3 ml of PBS(Ca^2+^/Mg^2+^) so that the buffered solution reaches the top of the wells.

### Spectroscopic analysis of the gas phase

In order to determine the presence of air impurities (N_2_, O_2_, H_2_O) in the plasma jet outside the quartz tube, optical emission spectroscopy was performed. The light emitted by the plasma jet was collected by a 10 cm focal length optical lens and its intensity detected with a 75 cm focal length spectrometer (Acton SP2750 with a 1800 grooves per mm grating blazed at 500nm) coupled with a 1340 pixel detector (Pixis from Roper Scientific). The emission spectra of the molecular bands of OH (at around 309 nm), N_2_(C) (Second Positive System at around 337 nm) and N_2_^+^(First Negative System at around 391 nm) and the atomic lines of He (at around 706 nm) and O (at around 777 nm) were recorded and normalized to the time of acquisition.

### Sensitivity of cells to H_2_O_2_

Cells at 50 to 70% confluence in 12-well plates were washed 2 times with PBS(Ca^2+^/Mg^2+^) and then exposed to 500 μl of PBS(Ca^2+^/Mg^2+^) containing increasing concentration of H_2_O_2_. The plates were left at room temperature for 1 h protected from light. Thereafter, 2.5 ml of DMEM containing 10% FCS, P/S 1X and L-gln 1X were added to the cells, and the plates were incubated at 37 °C and 5% CO_2_ in a humidified atmosphere for 24 h.

### Cell viability assay

To assess for the cell viability, the cell culture medium was removed from the plates, the cells washed once with DMEM without phenol red and covered with 500 μl of DMEM without phenol red containing 0.5 mg/ml thiazolyl blue tetrazolium bromide (MTT) (Sigma-Aldrich). The cells were incubated 2–3 h at 37 °C until purple precipitate was visible. The resulting intracellular purple formazan was then solubilized in the dark for 2 h in isopropanol 95%/0.4 N HCl. Spectrophotometric quantification was performed at 470 nm.

### Quantification of hydrogen peroxide (H_2_O_2_) in PBS(Ca^2+^/Mg^2+^) using sodium orthovanadate (Na_3_VO_4_) or titanium(IV) oxysulfate (TiOSO_4_)

The concentration of H_2_O_2_ in untreated and plasma-treated PBS(Ca^2+^/Mg^2+^) was determined using two methods. In the first method, H_2_O_2_ reacts with sodium orthovanadate to produce pervanadate, which is colourless^32^. In the second method, H_2_O_2_ reacts with titanium oxysulfate to produce pertitanic acid, which is yellow^33^,^34^. The formation of each product is detected spectrophotometrically. For the establishment of H_2_O_2_ standard curves by Na_3_VO_4_-based assay (method 1), serial dilutions of H_2_O_2_ were prepared in 500 μl of PBS(Ca^2+^/Mg^2+^), and Na_3_VO_4_ was added to a final concentration of 1 mM. For the establishment of H_2_O_2_ standard curves by TiOSO_4_-based assay (method 2), serial dilutions of H_2_O_2_ were prepared in 400 μl of PBS(Ca^2+^/Mg^2+^), 15 μl of 200 mM NaN_3_ were added and then 200 μl of 2% TiOSO_4_ diluted in 3 M H_2_SO_4_. NaN_3_ is used to scavenge nitrites and other ROS that can interfere with TiOSO4. For the determination of H_2_O_2_ concentration in plasma-treated PBS(Ca^2+^/Mg^2+^) by the method 1, 500 μl of PBS(Ca^2+^/Mg^2+^) containing (direct treatment) or not (indirect treatment) 1 mM Na_3_VO_4_ were exposed to He plasma for various times. For indirect treatment, Na_3_VO_4_ was added post treatment to plasma-treated PBS(Ca^2+^/Mg^2+^) from a stock solution at 200 mM. For the determination of H_2_O_2_ concentration in plasma-treated PBS(Ca^2+^/Mg^2+^) by the method 2, 500 μl of PBS(Ca^2+^/Mg^2+^) were exposed to He plasma for various times. Thereafter, 400 μl of plasma-treated PBS were mixed to 15 μl of 200 mM NaN_3_ and 200 μl of 2% TiOSO_4_ diluted in 3 M H_2_SO_4_. The samples were incubated protected from light for 30 min at room temperature to allow the reaction to occur, and the absorbance was measured at 260 and 270 nm (method 1) or at 407 nm (method 2). All reagents (Na_3_VO_4_, TiOSO_4_, H_2_O_2_ and NaN_3_) were from Sigma-Aldrich.

### Quantification of nitrite (NO_2_) and nitrate (NO_3_) in PBS(Ca^2+^/Mg^2+^)

The quantification of nitrite and nitrate was performed using the nitrate/nitrite colorimetric assay kit (Cayman) according to the supplier’s instructions.

### Measurements of pH in PBS(Ca^2+^/Mg^2+^)

The pH of untreated and treated buffered solutions was taken using a SevenEasy™ pH meter S20 fitted with a InLab^®^ Micro electrode (Mettler Toledo).

### Spectroscopic measurements of the liquid phase

All optical densities were recorded at room temperature in a double beam spectrophotometer (UVIKON XS, SECOMAM^®^, Servilab, France) using quartz cuvettes with a light path of 10 mm (Hellma). Known concentrations of H_2_O_2_, NaNO_2_ and NaNO_3_ were prepared in PBS(Ca^2+^/Mg^2+^). NaNO_2_ and NaNO_3_ were from Sigma-Aldrich.

### Statistical analysis

Results were plotted using a Microsoft Excel software as mean ± standard deviation. Student t-test was used to check the statistical significance (*p < 0.05, **p < 0.01, ***p < 0.001).

## Results

### Setting up of an assay to measure high concentration of H_2_O_2_ in plasma-treated PBS(Ca^2+^/Mg^2+^)

**Figure 2:**
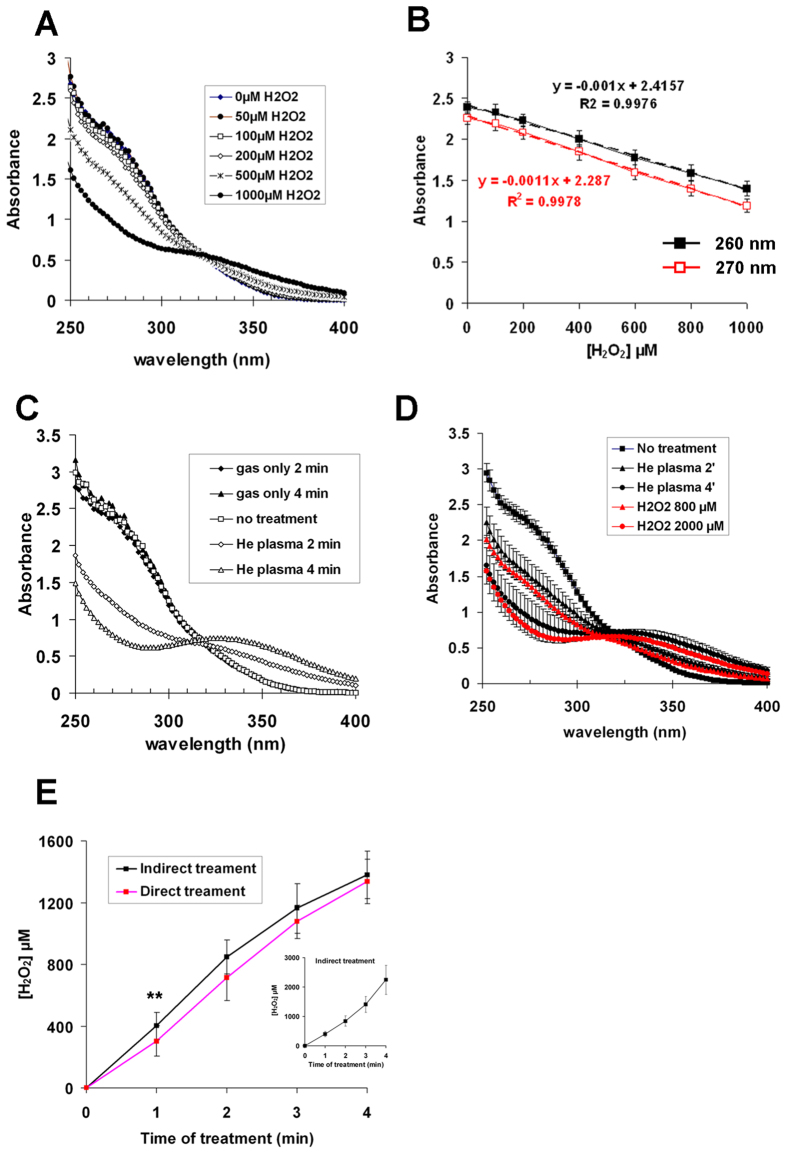
Quantification of H_2_O_2_ produced in PBS(Ca^2+^/Mg^2+^) by He plasma. (**A**) Absorption spectra between 250 and 400 nm of 1 mM Na_3_VO_4_ solutions in PBS(Ca^2+^/Mg^2+^) and incubated with increasing concentration of H_2_O_2_. (**B**) Correlation between the change in the optical density at 260 nm and 270 nm of 1 mM Na_3_VO_4_ solutions and the concentration of H_2_O_2_. The data are the mean ± sd of 12 independent experiments. The equations and correlation coefficients in black and red were derived from the linear regression of the values at 260 and 270 nm, respectively. (**C**) Absorption spectra between 250 and 400 nm of 1 mM Na_3_VO_4_ solutions in PBS(Ca^2+^/Mg^2+^) exposed to He gas or He plasma for 2 and 4 min. (**D**) Comparison between the absorption spectra of H_2_O_2_ solutions at 800 μM and 2 mM and the absorption spectra of plasma-activated PBS(Ca^2+^/Mg^2+^) after 2 and 4 min of treatment. The spectra are the average ± SD of 3 independent experiments. (**E**) Solutions of PBS(Ca^2+^/Mg^2+^) containing (direct treatment) or not (indirect treatment) 1 mM Na_3_VO_4_ were exposed to He plasma for 1, 2, 3, or 4 min. For indirect treatment, Na_3_VO_4_ was then added to plasma-treated PBS. The optical density of each solution was recorded at 260 and 270 nm, and the concentration of H_2_O_2_ determined using equations shown in panel B. The data are the mean ± SD of 12 independent experiments (*t-test* **p < 0.01). Insert: Solutions of PBS(Ca^2+^/Mg^2+^) were exposed to He plasma and were diluted 2x, 4x and 8x before adding Na_3_VO_4_. The concentration of H_2_O_2_ in each solution was determined as mentioned above by taking into account the dilution factors. The data are the mean ± SD of 9 independent experiments. For the experiments described in the panels C, D, E and F, the He flow was set to 50 sccm and the output voltage to 8 kV.

There are compelling evidences in the literature that plasma-induced liquid H_2_O_2_ plays a major role in the cellular toxicity of plasma-treated aqueous solutions^14^,^24^,^25^,^26^,^27^,^28^. Therefore, we wanted to precisely determine the concentration of H_2_O_2_ induced by our He plasma jet in a very simple buffer, phosphate buffered saline (PBS) containing Ca^2+^ and Mg^2+^, named PBS(Ca^2+^/Mg^2+^) hereafter. As the cells are exposed for one hour to PBS, we add the cations Ca^2+^ and Mg^2+^ as they contribute to maintain cell adhesion^35^. It has been shown that H_2_O_2_ can react in solution with Na_3_VO_4_ to yield pervanadate^32^. Therefore, we based our assay on a change of the absorbance of Na_3_VO_4_ upon its oxidation by H_2_O_2_. At first, we recorded the absorbance of different concentrations of Na_3_VO_4_ in PBS(Ca^2+^/Mg^2+^) between 200 and 400 nm, and found a concentration dependent increase of the optical density (O.D.) (Figure S2). For a concentration of 1 mM Na_3_VO_4_, the O.D. below 250 nm were higher than 3, closed to the maximum of the measurement range of the spectrophotometer (±3.5). Then, we prepared solutions of 1 mM Na_3_VO_4_ in 500 μl of PBS(Ca^2+^/Mg^2+^) and added increasing concentrations of H_2_O_2_. We observed a concentration-dependent decrease of the absorbance of Na_3_VO_4_ in the spectral range 250–320 nm and a slight increase in the range 320–400 nm (Fig. 2A). Based on these results, we focused on the change of O.D. at 260 and 270 nm. By repeating the measurements several times, we obtained a linear correlation between the change of O.D. at both wavelengths and the H_2_O_2_ concentration (Fig. 2B). Note that these correlations are true for concentrations of H_2_O_2_ ≤ 1 mM. We then exposed 1 mM Na_3_VO_4_ in PBS(Ca^2+^/Mg^2+^) to either a flow of He or a He plasma for 2 and 4 min, and we recorded the absorbance of the solutions between 250 and 400 nm. While the absorption spectrum of an untreated solution of 1 mM Na_3_VO_4_ was identical to those of the solutions only exposed to the He gas, we observed a time-dependent change of the absorbance of plasma-treated solutions (Fig. 2C). Interestingly, the absorption spectra obtained after 2 and 4 min of plasma treatment resemble those obtained after 800 and 2000 μM of H_2_O_2_ treatment, respectively (Fig. 2D). Because plasma treatment also leads to the formation of nitrite (NO_2_^−^) and nitrate (NO_3_^−^) in solution^16^,^17^, we checked that there was no change in the absorbance at 260 and 270 nm of 1 mM Na_3_VO_4_ incubated in the presence of either NaNO_2_ or NaNO_3_ for concentrations up to 3 mM (data not shown). Collectively, these results strongly support H_2_O_2_ as the major plasma-induced ROS that interact in solution with Na_3_VO_4_.

To confirm this hypothesis, 500 μl of PBS(Ca^2+^/Mg^2+^) containing (direct treatment) or not (indirect treatment) 1 mM Na_3_VO_4_ were exposed to He plasma at a gas flow of 50 sccm for different times of treatment up to 4 min. For the indirect treatment, Na_3_VO_4_ was added post treatment. We recorded the absorbance at 260 and 270 nm of the treated solutions, and used the equations shown in Fig. 2B to determine the concentration of the plasma-induced H_2_O_2_. We noticed that the concentration of H_2_O_2_ at a given time was identical in both conditions (Fig. 2E), suggesting that short-lived RONS produced in solution do not play a role in the reaction with Na_3_VO_4_. These results confirm that H_2_O_2_ is the major plasma-induced ROS that interact with Na_3_VO_4_. We also observed that the concentration of H_2_O_2_ increases almost linearly with the time of treatment to inflect at 4 min (Fig. 2E). This inflection is likely due to the non-linearity of the response for H_2_O_2_ concentration >1 mM (see Fig. 2B), and it was not observed if the plasma-treated solutions of PBS(Ca^2+^/Mg^2+^) were diluted before adding Na_3_VO_4_ (insert of Fig. 2E). From the data presented in Fig. 2E, we determined that about 400 μM of H_2_O_2_ are produced per minute of He plasma treatment at a gas flow of 50 sccm.

We also measured H_2_O_2_ concentration using titanium oxysulfate solution (TiOSO_4_)^33^,^34^. By this method, we found that about 300 μM of H_2_O_2_ are produced per minute of He plasma treatment at a gas flow of 50 sccm (Figure S3). Together, these results demonstrate that the concentration of H_2_O_2_ produced in PBS(Ca^2+^/Mg^2+^) by our He plasma device can range from a few hundred micromolar to a few millimolar, according to the time of treatment.

### More Nitrites than Nitrates are produced by He plasma

**Figure 3:**
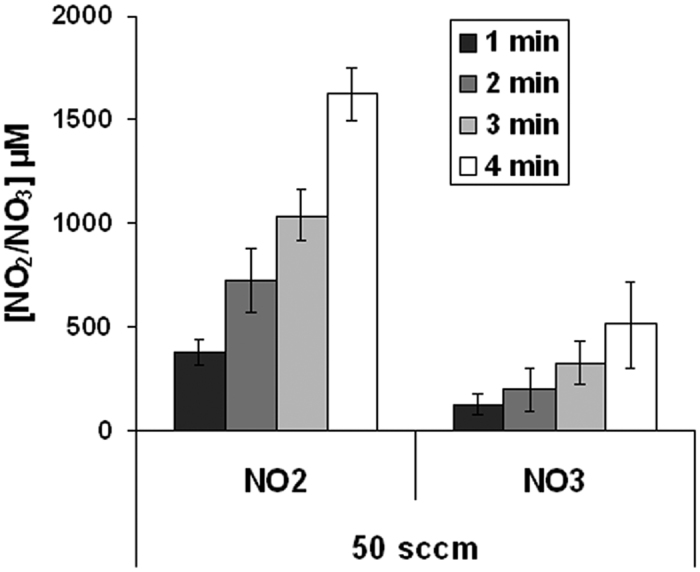
Quantification of NO_2_^−^ and NO_3_^−^ produced by He plasma. Solutions of PBS(Ca^2+^/Mg^2+^) were exposed to He plasma for 1, 2, 3, or 4 min and the concentration of NO_2_^−^ and NO_3_^−^ determined as described in Material and Methods. Note that NO_2_^−^ and NO_3_^−^ were not detectable in untreated (0 min) PBS(Ca^2+^/Mg^2+^). The data are the mean ± SD of 3 independent experiments. The He flow was set to 50 sccm and the output voltage to 8 kV.

To quantify NO_2_^−^ and NO_3_^−^ produced in the buffer solution by He plasma, 500 μl of PBS(Ca^2+^/Mg^2+^) were exposed to He plasma for 1, 2, 3 and 4 min and the amount of NO_2_^−^ and NO_3_^−^ was quantified using a colorimetric assay kit, as described in the Material and Methods section. We found a time-dependent increase of the concentration of each compound, NO_2_^−^ concentration being higher than the NO_3_^−^ concentration (Fig. 3). From these experiments, we determined that about 400 μM of NO_2_^−^ and 100 μM of NO_3_^−^ are produced in PBS(Ca^2+^/Mg^2+^) per minute of He plasma treatment at a gas flow of 50 sccm.

### H_2_O_2_, NO_2_^−^ and NO_3_^−^ represent the major species produced in PBS(Ca^2+^/Mg^2+^) by He plasma

**Figure 4:**
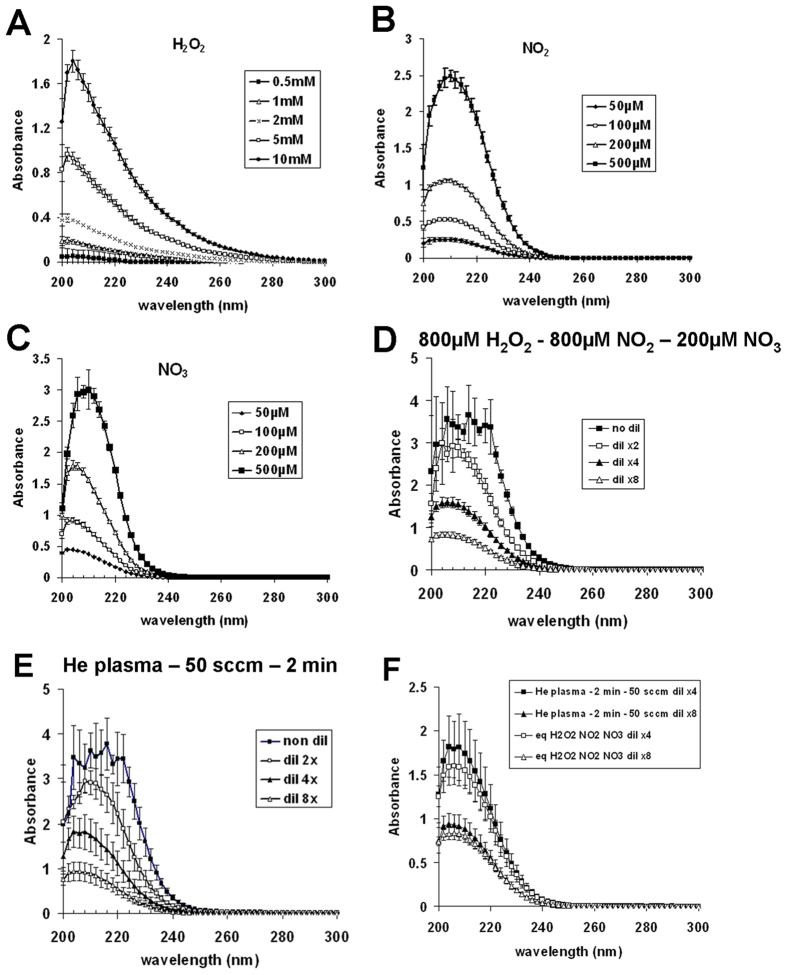
The absorption spectra of a mixture of H_2_O_2_, NO_2_^−^ and NO3^−^ match the absorption spectra of plasma-activated PBS(Ca^2+^/Mg^2+^). Absorption spectra between 200 and 300 nm of different concentrations of H_2_O_2_ (**A**), NO_2_^**−**^ (**B**) and NO_3_^−^ (**C**) prepared in PBS(Ca^2+^/Mg^2+^). The data are the mean ± SD of 5 (H_2_O_2_) and 4 (NO_3_^−^ and NO_2_^−^) independent experiments. (**D**) Absorption spectra of a mixture of 800 μM H_2_O_2_, 800 μM NO_2_^−^ and 200 μM NO_3_^−^ prepared in PBS(Ca^2+^/Mg^2+^). The data are the mean ± SD of 4 independent experiments. (**E**) 500 μl of PBS(Ca^2+^/Mg^2+^) were exposed to He plasma at a flow rate of 50 sccm for 2 min, and the absorption spectra were recorded between 200 and 300 nm. The data are the mean ± SD of 3 independent experiments. (**F**) Comparison between the absorption spectra of a mixture of 800 μM H_2_O_2_, 800 μM NO_2_^−^ and 200 μM NO_3_^−^ (curves shown in panel D) and the absorption spectra of plasma-activated PBS(Ca^2+^/Mg^2+^) (curves shown in panel E). As indicated in the panels D, E, and F, the solutions containing H_2_O_2_, NO_2_^−^ and NO_3_^−^ or plasma-activated PBS(Ca^2+^/Mg^2+^) were diluted 2x, 4x, or 8x in PBS(Ca^2+^/Mg^2+^) before the spectroscopic measurements.

To evaluate if the chemical modifications in PBS(Ca^2+^/Mg^2+^) can be attributed essentially to the formation of H_2_O_2_, NO_2_^−^ and NO_3_^−^ following plasma treatment, we performed UV spectrum analysis^36^,^37^. At first we recorded the absorption spectra in PBS(Ca^2+^/Mg^2+^) of each of these compounds at known concentrations. For NO_2_^−^ and NO_3_^−^, we used stock solutions of NaNO_2_ and NaNO_3_, respectively. We found that H_2_O_2_ poorly absorbs between 200 and 300 nm, with a maximum absorbance around 204 nm (Fig. 4A). Indeed, a 10 mM solution of H_2_O_2_ has an absorbance at 204 nm of 1.8 ± 0.1. In marked contrast, both NaNO_2_ and NaNO_3_ solutions strongly absorb between 200 and 250 nm, but not between 250 and 300 nm, with a maximum of absorbance at 210 nm (A210nm) (Fig. 4B,C). For example, A210nm = 2.48 ± 0.08 for a solution of NaNO_2_ at 0.5 mM, and A210nm = 1.56 ± 0.04 for a solution of NaNO_3_ at 0.2 mM (Fig. 4B,C).

As we previously demonstrated that approximately 400 μM of H_2_O_2_, 400 μM of NO_2_^−^ and 100 μM of NO_3_^−^ are generated per minute of He plasma treatment at a gas flow of 50 sccm (see above), we then looked at the absorbance of a mixed solution of 800 μM of H_2_O_2_, 800 μM of NO_2_^−^ and 200 μM of NO_3_^−^ (Fig. 4D). As these concentrations are expected to be produced in PBS(Ca^2+^/Mg^2+^) by He plasma after 2 min of treatment, we also recorded the absorbance of plasma-activated PBS(Ca^2+^/Mg^2+^) at such conditions (Fig. 4E). Because 800 μM of NO_2_^−^ alone gives rise to a A_210nm_ above the limits of the measurement range of the spectrophotometer, serial dilutions (dil 2x, 4x and 8x) were performed. As shown in Fig. 4D,E, the absorption spectra of plasma-activated PBS(Ca^2+^/Mg^2+^) were very similar to the absorption spectra of a mixed solution of 800 μM of H_2_O_2_, 800 μM of NO_2_^−^ and 200 μM of NO_3_^−^. To confirm these results, we superimposed the absorption spectra (dil x4 and x8) of plasma-activated PBS(Ca^2+^/Mg^2+^) and the absorption spectra (dil x4 and x8) of a mixed solution of 800 μM of H_2_O_2_, 800 μM of NO_2_^−^ and 200 μM of NO_3_^−^ (Fig. 4F). Indeed, for each dilution, the two absorption spectra were very similar suggesting that the three main long-lived species generated in PBS(Ca^2+^/Mg^2+^) by He plasma are H_2_O_2_, NO_2_^−^ and NO_3_^−^.

### Plasma-induced liquid H_2_O_2_ cannot account alone for the toxicity of plasma-activated PBS(Ca^2+^/Mg^2+^)

**Figure 5:**
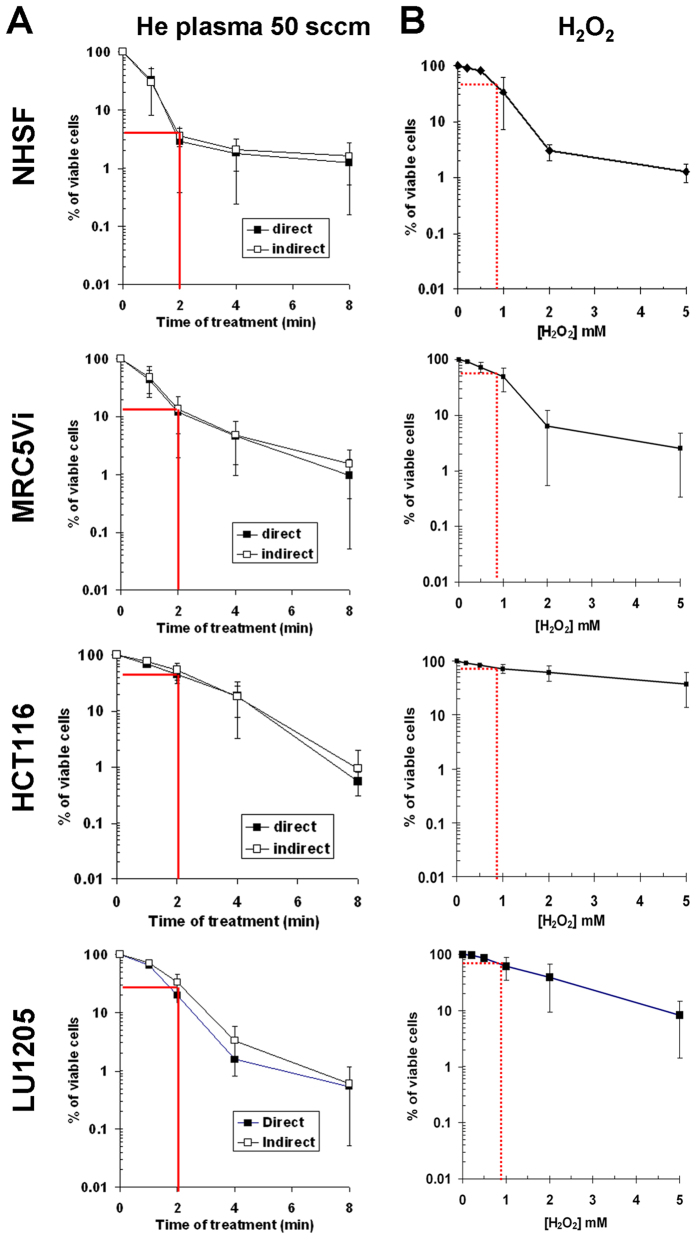
The concentration of plasma-induced H_2_O_2_ in PBS(Ca^2+^/Mg^2+^) does not fully explain the sensitivity of cells to He plasma. (**A**) Normal (MRC5Vi and NHSF) and tumour cells (HCT116 and Lu1205) were exposed in PBS(Ca^2+^/Mg^2+^) to He plasma (direct treatment) or to plasma-activated PBS(Ca^2+^/Mg^2+^) (indirect treatment) for 1, 2, 4 and 8 min, as described in Material and Methods. The He flow was set to 50 sccm and the output voltage to 8 kV. (**B**) The same cells were exposed to increasing concentration of H_2_O_2_ in PBS(Ca^2+^/Mg^2+^). The cell viability assay was performed 24 h post treatment. The data are the average ± SD of 3 to 4 independent experiments (He plasma treatment, *t-test* p > 0.05) and 5 to 8 independent experiments (H_2_O_2_ treatment). The continuous and dashed red lines indicate the percentage of viable cells after 2 min of He plasma treatment and after 800 μM of H_2_O_2_, respectively.

To assess the toxicity of He plasma at a gas flow of 50 sccm, we used normal primary skin fibroblasts (NHSF), normal transformed lung fibroblasts (MRC5Vi), human colon cancer cells (HCT116), and human melanoma cells (Lu1205). The cells were exposed directly or indirectly to He plasma for different times of treatment, and the cell viability was measured 24 hours post treatment. We observed for the four types of cells, a decrease in the % of cell viability as a function of the treatment time (Fig. 5A). Moreover, and as previously reported^14^,^19^,^22^, we did not observe a difference between the two modes of treatment (*i.e*. direct versus indirect) suggesting that the cytotoxicity of He plasma is essentially due to plasma-induced long-lived species in solution (Fig. 5A). The two tumour cell lines tested in this study (HCT116 and Lu1205) were slightly more resistant to the toxic effect of He plasma than the two normal cell lines (NHSF and MRC5Vi) especially for short (<4 min) treatment times (Fig. 5A).

As plasma-induced liquid H_2_O_2_ is a key ROS involved in the toxicity of several cold atmospheric plasmas^14^,^22^,^28^, we then measured the cytotoxicity of known concentrations of H_2_O_2_ with respect to the four cell types. Although we observed a concentration-dependent cell death for all cell types, the two cancer cell lines (HCT116 and Lu1205) were more resistant to H_2_O_2_-induced cell death than the two normal cells (NHSF and MRC5Vi) (Fig. 5B). This behaviour resembles to that observed after plasma treatment (Fig. 5A,B), suggesting that H_2_O_2_ plays a central role in the cellular toxicity of He plasma. However, if we consider a concentration of H_2_O_2_ of 800 μM, which is induced in PBS(Ca^2+^/Mg^2+^) after 2 min of He plasma (see Fig. 2), the % of viable cells, for the four cell types, is higher after a H_2_O_2_ treatment compared to a He plasma treatment (Fig. 5A,B). Indeed, the % of viable cells in response to 800 μM of H_2_O_2_ compared to 2 min of He plasma treatment was about 4% compared to 45% for NHSF, 15% compared to 55% for MRC5Vi, 45% compared to 70% for HCT116, and 30% compare to 70% for Lu1205. At longer times of treatment by He plasma (*i.e*. ≥4 min), the % of viable NHSF and MRC5Vi cells is almost identical to those obtained at the equivalent H_2_O_2_ concentration (*i.e*. ≥1.6 mM) (Fig. 5). In contrast, the % of viable HCT116 and Lu1205 cells is always higher in response to a treatment of H_2_O_2_ than to a He plasma treatment, in the concentration and time range considered in this study (Fig. 5). These data strongly suggest that other RONS than H_2_O_2_ also contribute to the toxicity of the He plasma.

### The concentrations of plasma-induced H_2_O_2_, NO_2_^−^ and NO_3_^−^ are lower at a higher gas flow

**Figure 6:**
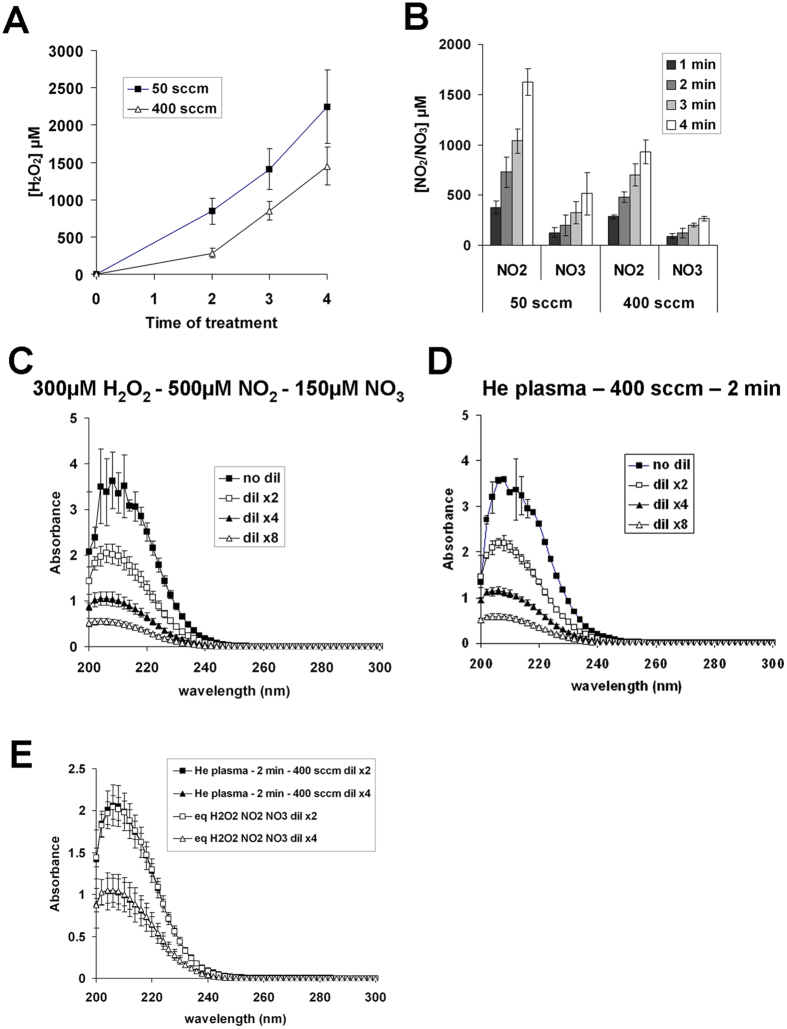
Increasing the He flow decreases the concentration of plasma-induced H_2_O_2_, NO_2_^−^ and NO_3_^−^. 500 μl of PBS(Ca^2+^/Mg^2+^) per well in 12 well plate were exposed to 50 or 400 sccm of He plasma for 1, 2 and 4 min. The concentration of H_2_O_2_ (**A**) and NO_2_/NO_3_ (**B**) were determined using the Na_3_VO_4_-based method and the nitrate/nitrite colorimetric assay kit, respectively. The data are the mean ± SD of 5 (H_2_O_2_) and 4 (NO_3_^−^ and NO_2_^−^) independent experiments. (**C**) Absorption spectra of a mixture of 300 μM H_2_O_2_, 500 μM NO_2_^−^ and 150 μM NO_3_^−^ prepared in PBS(Ca^2+^/Mg^2+^). The data are the mean ± SD of 4 independent experiments. (**D**) 500 μl of PBS(Ca^2+^/Mg^2+^) were exposed to He plasma at a flow rate of 400 sccm for 2 min, and the absorption spectra were recorded between 200 and 300 nm. The data are the mean ± SD of 3 independent experiments. (**E**) Comparison between the absorption spectra of a mixture of 300 μM H_2_O_2_, 500 μM NO_2_^−^ and 150 μM NO_3_^−^ (curves shown in panel C) and the absorption spectra of plasma-activated PBS(Ca^2+^/Mg^2+^) (curves shown in panel D). As indicated in the panels C, D, and E, the solutions containing H_2_O_2_, NO_2_^−^ and NO_3_^−^ or plasma-activated PBS(Ca^2+^/Mg^2+^) were diluted 2x, 4x, or 8x in PBS(Ca^2+^/Mg^2+^) before the spectroscopic measurements.

All the experiments described above were performed at a He gas flow of 50 sccm. To investigate the effect of the gas flow on the RONS induced in PBS(Ca^2+^/Mg^2+^), we determined the concentration of H_2_O_2_, NO_2_^−^ and NO_3_^−^ in the buffer solution after a He plasma treatment at a gas flow of 400 sccm. We found that the concentration of H_2_O_2_ (Fig. 6A) and of NO_2_^−^ and NO_3_^−^ (Fig. 6B) is lower at 400 sccm compared to 50 sccm. Indeed, after 2 min of He plasma treatment at 400 sccm, the concentration of H_2_O_2_ was about 300 μM (instead of 800 μM at 50 sccm), while the concentrations of NO_2_^−^ and NO_3_^−^ were about 500 μM and 150 μM, respectively (instead of 800 μM and 200 μM at 50 sccm). In order to verify if the absorption spectrum of a mixture of these RONS at these concentrations could fully reproduce the absorption spectrum of a solution of PBS(Ca^2+^/Mg^2+^) treated for 2 min with a He plasma at a gas flow of 400 sccm, we recorded and compared the absorption spectra of PBS(Ca^2+^/Mg^2+^) solution containing 300 μM of H_2_O_2_, 500 μM of NO_2_^−^ and 150 μM of NO_3_^−^ (Fig. 6C), and of plasma-activated PBS(Ca^2+^/Mg^2+^) after 2 min of treatment (Fig. 6D). The overlay of the spectra for each of the two conditions, at the same dilution factor (Fig. 6E), suggests that the mixture of these three species at the measured concentrations can adequately reproduce the chemistry generated in the buffer solution after 2 min of He plasma at a gas flow of 400 sccm.

### Plasma-induced liquid H_2_O_2_ cannot account alone for the toxicity of plasma-activated PBS(Ca^2+^/Mg^2+^) at a gas flow of 400 sccm

**Figure 7:**
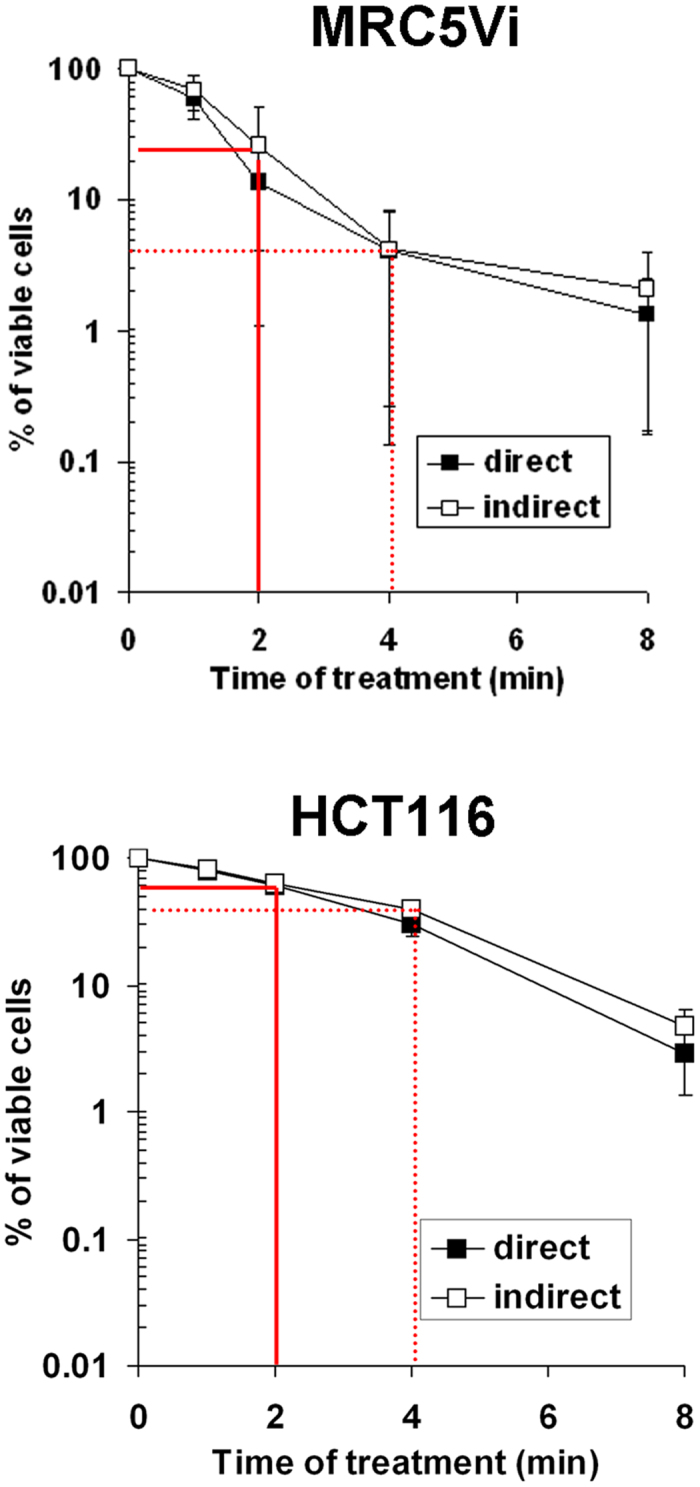
Percentage of viable cells after a He plasma treatment at a gas flow of 400 sccm. MRC5Vi and HCT116 cells were exposed in PBS(Ca^2+^/Mg^2+^) to He plasma (direct treatment) or to plasma-activated PBS(Ca^2+^/Mg^2+^) (indirect treatment) for 1, 2, 4 and 8 min. The He flow was set to 400 sccm and the output voltage to 8 kV. The cell viability assay was performed 24 h post treatment. The red lines indicate the percentage of viable cells after 2 min of He plasma treatment. The data are the average ± SD of 4 independent experiments (*t-test* p > 0.05)

We used MRC5Vi cells, as control of normal cells, and HCT116, as control of tumour cells to assess the role of H_2_O_2_ in the toxicity of the He plasma at a gas flow of 400 sccm. As reported above for a He plasma operating at a gas flow of 50 sccm (see Fig. 5A), the indirect treatment is as efficient as the direct treatment in inducing cell death also at a gas flow of 400 sccm (Fig. 7). Moreover, HCT116 cells were also found more resistant than MRC5Vi to the plasma treatment at a gas flow of 400 sccm (Fig. 7), thus confirming the results obtained at 50 sccm (see Fig. 5A). We showed that at a gas flow of 400 sccm, the He plasma generates about 300 μM H_2_O_2_ per min of treatment (see Fig. 6A). From the sensitivity of each cell line to H_2_O_2_ (see Fig. 5B), if the toxicity arises only from plasma-induced liquid H_2_O_2_, then the % of viable cells should range between 60% (for MRC5Vi) to 95% (for HCT116) after 2 min of He plasma treatment, and between 30% (for MRC5Vi) to 60% (for HCT116) after 4 min of He plasma treatment. We found that after 2 min of He plasma treatment, the % of viable cells was about 20% for MRC5Vi and 60% for HCT116, while after 4 min of treatment, these values dropped to 4% and 40%, respectively (Fig. 7). Therefore, the % of viability obtained after plasma treatment is lower than those determined after H_2_O_2_ treatment alone, demonstrating that H_2_O_2_ alone cannot account for the toxicity of plasma-activated PBS(Ca^2+^/Mg^2+^) at a gas flow of 400 sccm, as it was also observed at 50 sccm.

### NO_2_^−^ and H_2_O_2_ act synergistically to trigger cell death after plasma treatment

**Figure 8:**
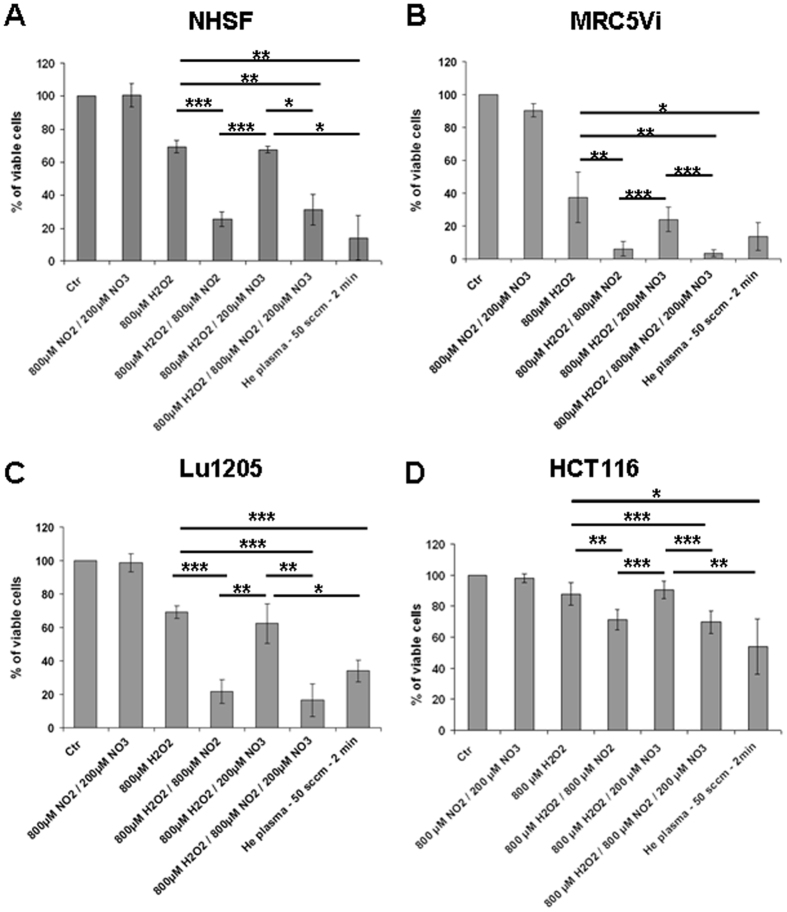
Synergic effect of H_2_O_2_ and NOs species on cell viability. NHSF (**A**), MRC5Vi (**B**), Lu1205 (**C**), and HCT116 (**D**) cells were untreated (Ctr) or exposed to a mixture of NO_2_^−^ and NO_3_^−^, H_2_O_2_ alone, a mixture of H_2_O_2_ and NO_2_^−^ or NO_3_^−^, or a mixture of H_2_O_2_, NO_2_^−^ and NO_3_^−^. The concentrations of H_2_O_2_, NO_2_^−^ and NO_3_^−^ were those determined after a He plasma treatment of 2 min at a gas flow of 50 sccm (see Fig. 3). The cell viability assay was performed 24 h post treatment. The data are the mean ± SD of 6 independent experiments. The cell viabilities after an indirect He plasma treatment at a gas flow of 50 sccm are also indicated.

So far, our results demonstrated that the three main species generated in PBS(Ca^2+^/Mg^2+^) by He plasma are H_2_O_2_, NO_2_^−^ and NO_3_^−^, and that H_2_O_2_ is essential, but not sufficient, to account for the toxicity of He plasma. These observations prompted us to investigate the role of NO_2_^−^ and NO_3_^−^ in the toxicity of plasma-activated PBS(Ca^2+^/Mg^2+^). To do so, NHSF, MRC5Vi, HCT116 and Lu1205 cells were exposed to different solutions of PBS(Ca^2+^/Mg^2+^) containing H_2_O_2_ and/or NO_2_^−^ and/or NO_3_^−^ at the concentrations obtained after 2 min of He plasma treatment at a gas flow of 50 sccm (*i.e*. 800 μM H_2_O_2_, 800 μM NO_2_^−^ and 200 NO_3_^−^). We show that in the range of concentrations used in this study, NO_2_^−^ and/or NO_3_^−^ are not toxic to the cells (Fig. 8 and Figure S4A), and that the sensitivity of each cell line to H_2_O_2_ treatment is not enhance by the addition of NO_3_^−^ (*t*-test p > 0.05) (Fig. 8). In contrast, a mixture of H_2_O_2_ and NO_2_^−^ triggered more cell death than H_2_O_2_ alone, and again the addition of NO_3_^−^ to H_2_O_2_/NO_2_^−^ mixture did not change the % of viable cells (*t*-test p > 0.05) suggesting that NO_3_^−^ does not contribute to cell death (Fig. 8). Finally, and most importantly, we found that the % of viable cells in response to a mixture of H_2_O_2_/NO_2_^−^ (or H_2_O_2_/NO_2_^−^/NO_3_^−^) is not statistically different to the % of viable cells in response to plasma-activated PBS(Ca^2+^/Mg^2+^) (*t*-test p > 0.05) (Fig. 8). Because H_2_O_2_ can react with NO_2_^−^ in weakly acid to acid aqueous solutions to form peroxinitric acid^38^, we also monitored the pH of plasma-activated PBS(Ca^2+^/Mg^2+^) as a function of treatment time at a gas flow of 50 sccm. For comparison, we also checked the pH of PBS(Ca^2+^/Mg^2+^) containing a mixture of H_2_O_2_/NO_2_^−^/NO_3_^−^ corresponding to the concentrations expected after plasma treatment. We found a treatment time-dependent decrease of the pH of plasma-activated PBS(Ca^2+^/Mg^2+^) but not of reconstituted buffered solutions (Figure S4B). Indeed, a drop of the pH from 7.2 to 6 was observed after 8 min of treatment. Collectively, our results strongly suggest that plasma-induced-H_2_O_2_ and -NO_2_^−^ in PBS(Ca^2+^/Mg^2+^) act in synergy, possibly in part *via* the formation of peroxynitrite, to induce cell death.

### The concentration of plasma-induced H_2_O_2_, NO_2_^−^ and NO_3_^−^ in PBS(Ca^2+^/Mg^2+^) is decreased when pure oxygen is used as the shielding gas

**Figure 9:**
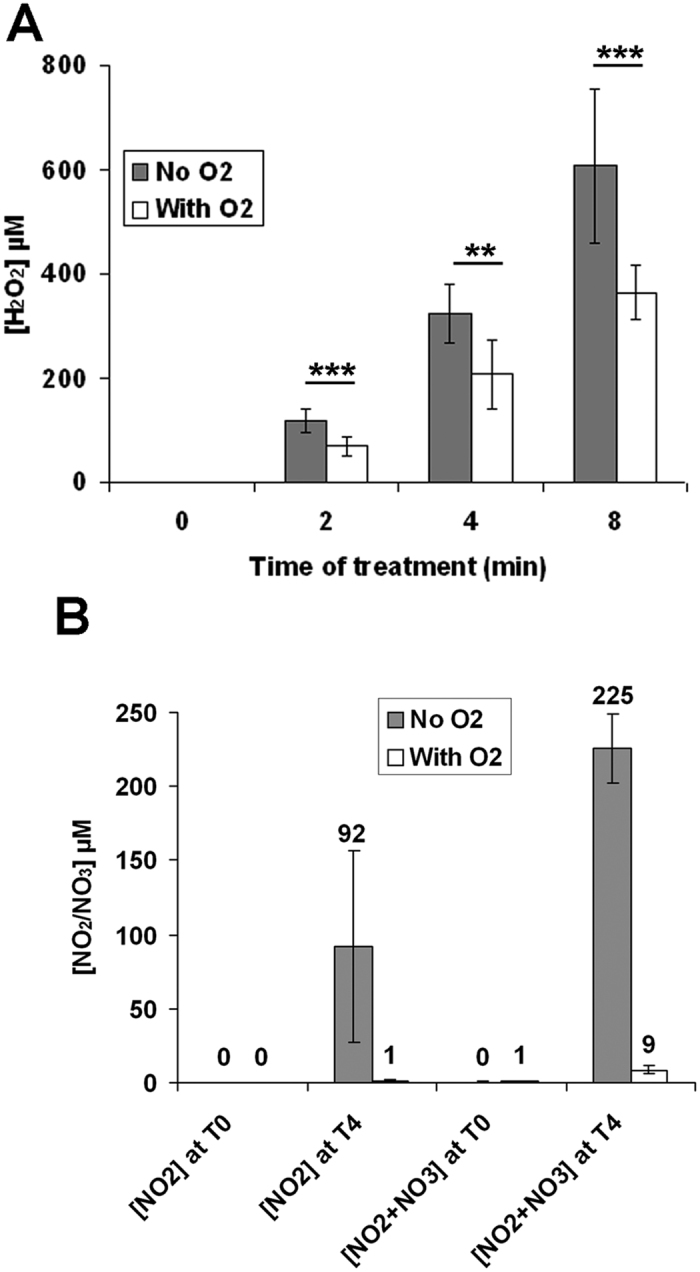
The concentrations of H_2_O_2_, NO_2_^−^ and NO3^−^ in plasma-treated PBS(Ca^2+^/Mg^2+^) are decreased in the presence of a shielding gas of pure oxygen. Three milliliters of PBS(Ca^2+^/Mg^2+^) were set per well in 12 well plates, and exposed to He plasma at a gas flow of 100 sccm for the indicated period of times, and at an output voltage of 5.5 kV. The treatments were performed in the presence (with O_2_) or absence (no O_2_) of a shielding gas of oxygen at a gas flow of 5 slm. (**A**) The concentration of plasma-induced H_2_O_2_ for each time point was determined by using the Na_3_VO_4_ assay and the TiOSO_4_ assay. (**B**) The concentrations of NO_2_^−^ and NO_3_^−^ after 4 min of plasma treatment were determined by the Griess assay.

**Figure 10:**
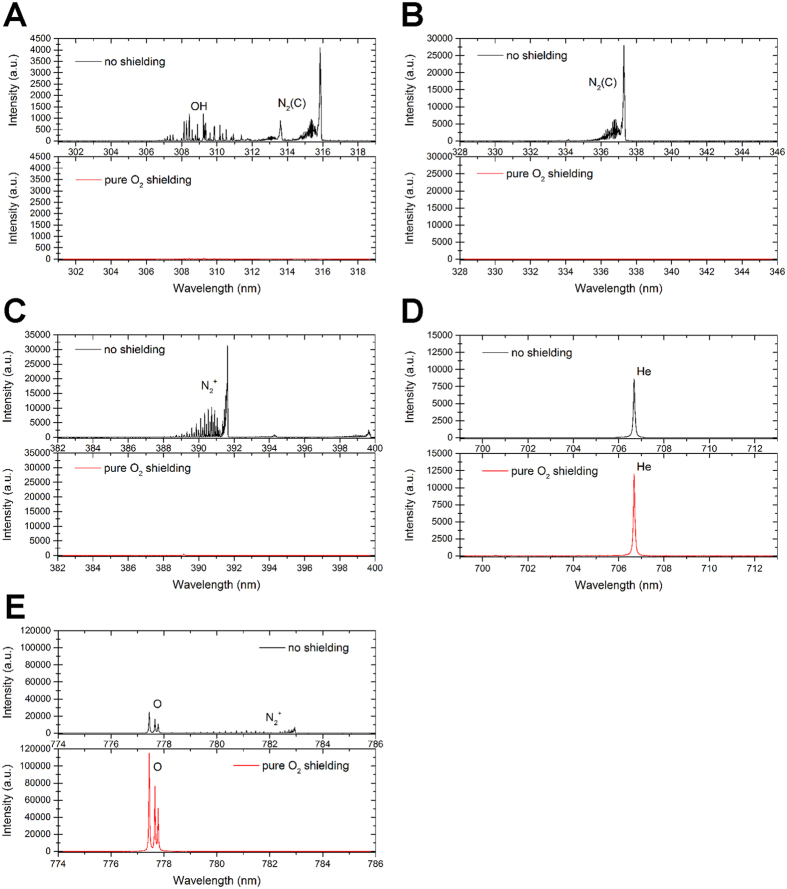
A shielding gas of pure oxygen strongly inhibits the presence of OH and N_2_ molecules in the gas phase. The emission spectra of the molecular bands of (**A**) OH (at around 309 nm), (**B**) N_2_(C) (Second Positive System at around 337 nm) and (**C**) N_2_^+^ (First Negative System at around 391 nm) and the atomic lines of (**D**) He (at around 706 nm) and (**E**) O (at around 777 nm) were recorded in the absence (no shielding) or presence (pure O_2_ shielding) of a shielding of pure O_2_. The gas flow of He was set to 100 sccm and the plasma is created by applying high voltage pulses with amplitude of 5.5 kV.

To assess for the role of atmospheric ambient air in the formation of plasma-induced RONS, the He plasma jet was shielded from the atmosphere (ambient air) by a gas of pure O_2_. As the experimental setup used for this specific study was slightly different to the one used so far (see Material and Method), at first we decided to measure the concentration of H_2_O_2_, NO_2_^−^ and NO_3_^−^ produced in these new experimental conditions. Using a He gas flow at 100 and 400 sccm, we found that the production of H_2_O_2_ was 63 and 35 μM per min, respectively (Figure S5A), while the production of total NOx (NO_2_^−^+NO_3_^−^) was 33 and 12 μM per min, respectively (Figure S5B). These values are lower than those measured with the other plasma jet (see Figs 2,3 and 6), but can be explained at least by the larger volume of treated PBS(Ca^2+^/Mg^2+^) used here (3 ml instead of 0.5 ml) and the lower output voltage (5.5 kV instead of 8 kV). Nevertheless, we found again that increasing the gas flow leads to lower the concentration of these RONS in the plasma-treated solution. Using a shielding gas of pure O_2_ surrounding the plasma jet, we found that after 4 min of treatment, the concentration of H_2_O_2_ drops by 36% (322 μM with ambient air to 206 μM with pure O_2_) (Fig. 9A) while the concentration of total NOx (NO_2_^−^ + NO_3_^−^) drops by 96% (226 μM with ambient air to 9 μM with pure O_2_) (Fig. 9B). These reductions of the production of H_2_O_2_ and NO_2_^−^/NO_3_^−^ follow the observed decrease of the light intensity emitted in the plasma jet by OH and N_2_(C)/N_2_^+^ of about 1 and 3 orders of magnitude when a shielding of pure O_2_ is applied (Fig. 10 and Table 1). Concomitantly, the shielding gas of pure O_2_ also prevented the acidification of the plasma-treated PBS(Ca^2+^/Mg^2+^) (Figure S6).

## Discussion

The application of cold atmospheric pressure plasmas (CAPPs) in cancer treatment is one of the main active fields of research in *Plasma Medicine*. The “proof-of-concept” has been largely demonstrated *in vitro* and to a lesser extent *in vivo* (for a recent review see ref. 3). Although the different groups working in this field used different plasma devices with different plasma chemistries and cell lines derived from different tumours^3^, all the different types of CAPPs were effective, indicating that the effects of plasma seem to be uniform and are not restricted to a particular type of tumour. One fundamental insight arising from all these studies is that plasma-induced changes in the liquid environment of the cells play a key role in plasma-cell interactions, and thus to the cell fate. As mentioned by D. Graves in 2012: “The successful development of plasma biomedicine applications will hinge in significant measure on controlling the actions of the RONS created in the plasma by generating only the species that are needed and delivering them to the right place at the right time in the right concentration”^16^. To date, it is unanimously recognized that RONS, among them H_2_O_2_, NO_2_^−^ and NO_3_^−^, are the central players in the antitumor activities of CAPPs^16^,^17^. The aim of this study was to precisely determine the concentration of each of these species in solution after a He plasma treatment and to address the following question: is the production of one or more of these species in solution sufficient to explain the cellular toxicity of the He plasma device?

At first, we would like to draw attention to the fact that it is difficult to strictly compare the measured concentration of each species obtained in one study (including ours) to other published studies insofar as different types of CAPPs and biological targets are used. Any parameters of the experimental setup (*e.g*. the nature of the gas, the gas flow, the applied voltage, the distance between the plasma and the solution, the composition of the solution, the volume of the solution …)^15^,^21^ play a role in the amount of plasma-induced RONS in solution. Hereafter are some selected examples regarding the concentration of plasma-induced H_2_O_2_ in different conditions: [H_2_O_2_] = 30 μM in 500 μl of MEM medium after 1 min of He plasma at a gas flow of 5 L/min^24^. [H_2_O_2_] = 6 μM in 300 μl of phenol-free RPMI 1640 medium after 1 min of He +0.25% O_2_ plasma at a gas flow of 8 L/min^22^; [H_2_O_2_] = 60 μM in 1 ml of phenol-free RPMI 1640 medium after 1 min of Ar plasma at a gas flow of 3 L/min^28^; [H_2_O_2_] = 32 μM in 5 ml of phenol-free RPMI 1640 medium after 1 min of Ar plasma at a gas flow of 3 L/min^39^; [H_2_O_2_] = 190 μM in 3 ml of PBS(Ca^2+^/Mg^2+^/Glucose) after 1 min of Ar plasma at a gas flow of 1.5 L/min.

Using two different assays (one based on Na_3_VO_4_ and the other on TiOSO_4_), we showed that in our standard experimental conditions–500 μl of PBS(Ca^2+^/Mg^2+^) exposed to a He plasma jet operated at 8 kV and at a gas flow of 50 sccm - around 400 μM of H_2_O_2_ are produced per min, a value which is quite high compare to those cited above. Yang *et al*. reported that the concentration of ROS measured after plasma treatment decreases with increasing the complexity of the targeted solution^22^. We carried out our experiments in a simple buffered solution [PBS(Ca^2+^/Mg^2+^)], which is devoid of amino acids, vitamins and other compounds, such as glucose or serum found in all cell culture media. The presence of some of these components in the cell culture media during plasma exposure might interfere with the formation of H_2_O_2_, or react with H_2_O_2_^21^. Furthermore, we used a very small He flow rate (50 sccm), when compared to most published data for which He flow rates of few liters per min were used^15^,^21^,^23^,^24^,^40^,^41^,^42^, and, as further discussed below in the text, we found that the concentration of H_2_O_2_ in solution is higher as the gas flow is lower.

We also found that the rate production of NO_2_^−^ and NO_3_^−^ is 400 μM and 100 μM per min, respectively, at a gas flow of 50 sccm. As for H_2_O_2_, the concentration of these RNS in solution is also highly dependent on the experimental setup [this study, see also^14^,^25^]. By looking at the absorption in the UV range (200–300 nm) of solutions of PBS(Ca^2+^/Mg^2+^) exposed to a He plasma jet and solutions of PBS(Ca^2+^/Mg^2+^) containing a mixture of H_2_O_2_, NO_2_^−^ and NO_3_^−^, we have been able to demonstrate that these species account for the main long-lived RONS induced by our plasma in the buffer solution. Aiming the understanding of how these species accumulate in solution during the He plasma treatment, we used a shielding gas of pure O_2_ isolating the plasma jet from the ambient air. We found that the surrounding atmosphere has a greater impact on the formation of NO_2_^−^ and NO_3_^−^ than on the formation of H_2_O_2_ in solution. These results are in good agreement with those published by Tresp *et al*. who used argon as the feeding gas^12^. In buffer solution, the dissociation of water molecules by energetic particles from the plasma can generate hydroxyl radicals that recombine to form H_2_O_2_^12^,^27^,^43^,^44^. The water molecules can already be present in the feeding gas (*e.g*. using a humidified feeding gas)^14^,^27^,^45^ or arise from the humidity in the ambient air, through which the plasma propagates, and from the water vapor evaporated from the water outer layer of the solution targeted with the plasma^44^. In our experimental conditions, we used as feeding gas Helium Alphagaz 2 that contains only traces of H_2_O_2_ (<0.5 ppm). Therefore, water molecules mainly arise from the ambient air, and at the gas/liquid interface. Using a shielding gas of pure O_2_, we showed that both pathways contribute almost equally to the liquid H_2_O_2_ induced in the buffer solution by the plasma. Concomitantly, we showed that the concentration of NO_2_^−^ and NO_3_^−^ drops drastically in the presence of the shielding gas of pure O_2_. This was expected as nitrites and nitrates are formed in plasma-treated buffer solutions through the dissolution of nitrogen oxides produced by gas-phase reactions of dissociated N_2_ and O_2_^46^. By increasing the He gas flow, we found a decrease of the concentration of the three species in the buffer solution. This should result from the fact that less air is admixed to the plasma jet channel when the gas flow is higher and, thus, less RONS are produced in the gas phase.

The effects of theses plasma-generated species on mammalian cells were investigated on four different cell types: 2 normal cell types (NHSF and MRC5Vi) and 2 cancer cell lines (HCT116 and Lu1205). We confirmed several published data showing that plasma-activated medium is as efficient as the direct treatment of cells in triggering cell death^14^,^18^,^19^,^20^,^21^,^22^,^23^,^24^. Although we did not investigate further the route leading to cell death, it is well documented that apoptosis (a programmed cell death) and necrosis (a non physiological process) are the two main cell death pathways that have been described after CAPP treatments^23^,^26^,^29^,^47^,^48^,^49^, and are likely involved in our study. Several investigators have shown that cancer cells are more susceptible to plasma-induced cell death than normal or healthy cells^20^,^41^,^50^,^51^. It was proposed that the distribution of the cells within the cell cycle may account for the higher susceptibility of cancer cells to CAPP treatment^52^. In a recent review, Yan *et al*. proposed that cancer cells tend to express more aquaporins on their cytoplasmic membranes, which may cause the H_2_O_2_ uptake speed in cancer cells to be faster than in normal cells^53^. However, these observations contrast with other published data^26^,^49^,^54^ and our data (this study) showing that the cancer cells are more resistant to CAPP treatment than the normal or healthy cell types. To explain such discrepancies, further investigations are required but it is necessary to consider several parameters such as the plasma device used in each experimental setup, the concentration of RONS produced in solution, the nature of the treated solution (*e.g*. PBS *versus* cell culture medium) and the type of targeted cells. Regarding this last point, an effective comparison between the responses of normal and cancer cells to CAPP treatment should be performed between cell lines derived from the same tissue^53^.

Our observations also confirm that, at least regarding cell death, long lifetime species such as H_2_O_2_ and NO_2_^−^ fully account for the toxicity of CAPPs. We have demonstrated that the sensitivity of the four cell lines to the He plasma treatment parallels their individual sensitivity to H_2_O_2_, thus pointing to H_2_O_2_ as a central player in plasma-induced oxidative stress^24^,^28^, and that the concomitant production of NO_2_^−^ exacerbates H_2_O_2_ toxicity. In weakly acid to acid solutions, peroxynitric acid can be formed by the interaction of NaNO_2_ and H_2_O_2_^38^,^45^,^55^. As the He plasma treatment led to acidification of PBS(Ca^2+^/Mg^2+^), it is reasonable to think that peroxynitrite is formed, especially at long treatment times. Peroxynitrite can induce both cellular apoptosis and necrosis depending on the production rates, endogenous antioxidant levels and exposure time^56^, and therefore could contribute to plasma-induced cell death. We propose that the ability of the cells to cope with these two RONS (H_2_O_2_/NO_2_^−^) is the major signal that triggers the cell fate in response to our He plasma device. Nonetheless, we cannot not exclude that others plasma-induced RONS, such as nitric oxide (NO), radical hydroxyl (HO.), superoxide anion (O_2_^−^)^29^,^57^,^58^ could contribute, to a less extent, to plasma toxicity. At the level of the cellular response, the control of the intracellular redox homeostasis^59^, the activation of MAPK pathways^25^,^60^, the down regulation of survival signal transduction pathway^18^, the epigenetic and cellular changes that are induced by CAPP in a cell type-specific manner^61^, the distribution of the cells within the cell cycle^52^, the expression of aquaporins^53^ are all endpoints to take into account to evaluate the effectiveness of CAPPs as a new antitumor strategy.

## Additional Information

**How to cite this article**: Girard, P.-M. *et al*. Synergistic Effect of H_2_O_2_ and NO_2_ in Cell Death Induced by Cold Atmospheric He Plasma. *Sci. Rep*. **6**, 29098; doi: 10.1038/srep29098 (2016).

## Supplementary Material

Supplementary Information

## Figures and Tables

**Table 1:** Ratio of the relative intensities of the light emission of the molecular bands of OH, N_2_(C) and N_2_^+^ and of the atomic lines of He and O from the plasma jet in the absence or presence of a shielding gas of pure O_2_.

Gas species	Ratio (no shielding/O_2_ shielding)
OH (309 nm)	33
N_2_(C) (337 nm)	2155
N_2_^+^ (391 nm)	1475
He (706 nm)	0.72
O (777 nm)	0.22

The data are derived from the experimental values measured from the emission spectra of each species shown in Fig. 10. Note that the emission intensity of the molecular bands of OH, N_2_(C) and N_2_^+^ drop drastically in the presence of the shielding gas of pure O_2_.
