# Supplementary material for: Synergistic Effect of H2O2 and NO2 in Cell Death Induced by Cold Atmospheric He Plasma
Source: Sci Rep. 2016 Jul 1;6:29098. doi: 10.1038/srep29098 (PMC4929573; doi:10.1038/srep29098)
Supplement: Supplementary Information [file srep29098-s1.pdf]

Synergistic Effect of H_2O_2 and NO_2 in Cell Death Induced by Cold Atmospheric He Plasma

Pierre-Marie Girard[1,2,§], Atousa Arbabian[3], Michel Fleury[3], Gérard Bauville[3], Vincent Puech[3], Marie Dutreix[1,2], and Joao Santos Sousa[3,§]

[1] Institut Curie, PSL Research University, CNRS UMR3347, INSERM U1021, 91405, Orsay, France.

[2] Université Paris-Sud, Université Paris-Saclay, rue Georges Clémenceau, 91405 Orsay, France

[3] LPGP, CNRS, Université Paris-Sud, Université Paris-Saclay, 91405 Orsay, France

[§] Corresponding authors: Pierre-Marie Girard, Institut Curie, PSL Research University, CNRS UMR 3347, INSERM U1021, 91405 Orsay, France, pierre-marie.girard@curie.fr. Joao Santos Sousa, LPGP, CNRS, Univ. Paris-Sud, Université Paris-Saclay, 91405 Orsay, joao.santos-sousa@u-psud.fr

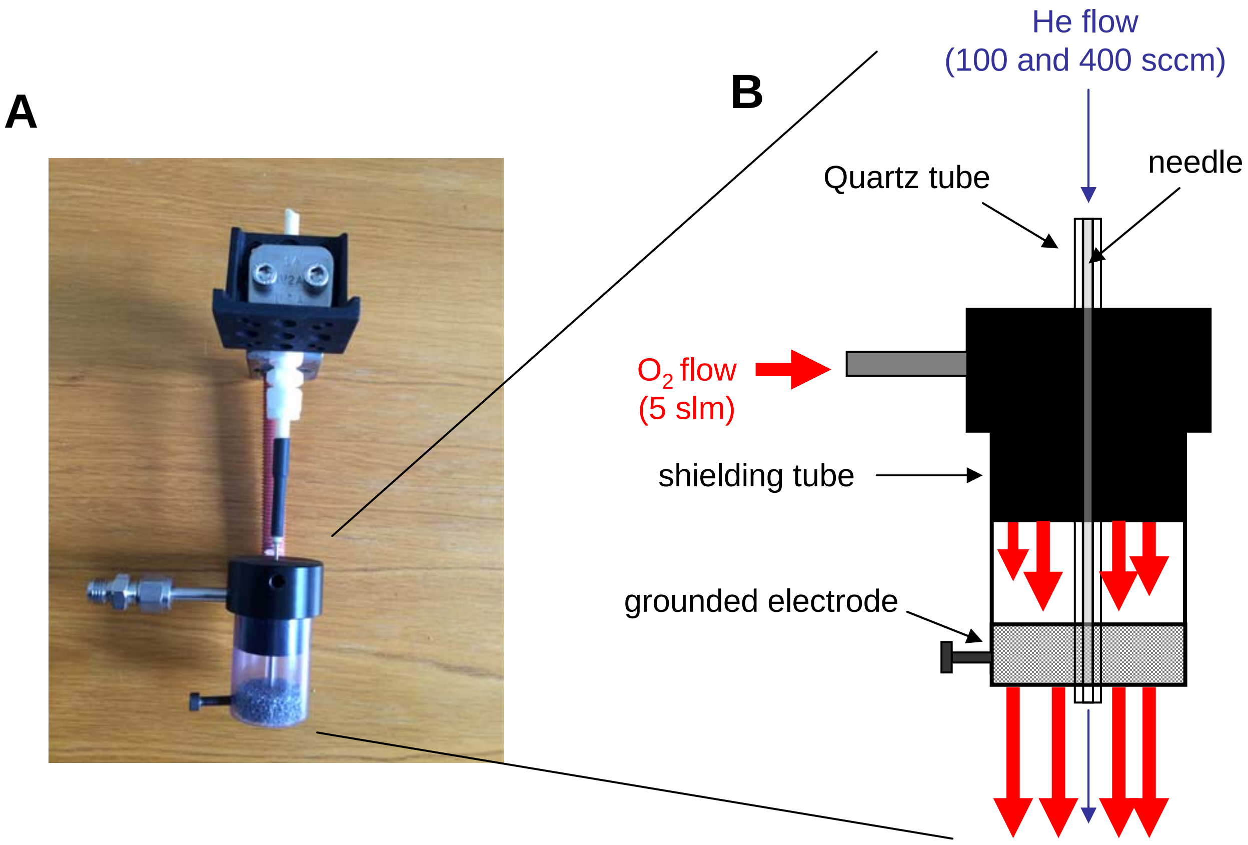
A
B
He flow
(100 and 400 sccm)
Quartz tube
needle
O_2 flow
(5 slm)
shielding tube
grounded electrode

Figure S1

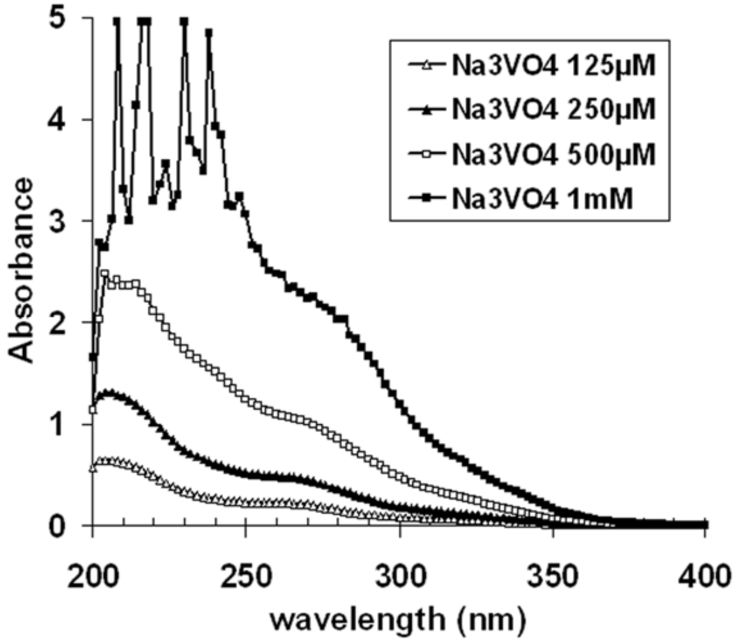

Absorbance
5
4
3
2
1
0
200
250
300
350
400
wavelength (nm)
Na3VO4 125µM
Na3VO4 250µM
Na3VO4 500µM
Na3VO4 1mM

Figure S2

A

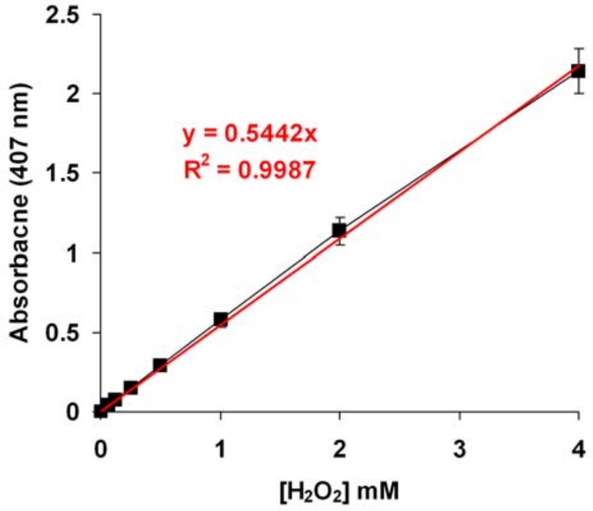

2.5
2
1.5
1
0.5
0
Absorbacne (407 nm)
y = 0.5442x
R^2 = 0.9987
0
1
2
3
4
[H_2O_2] mM

B

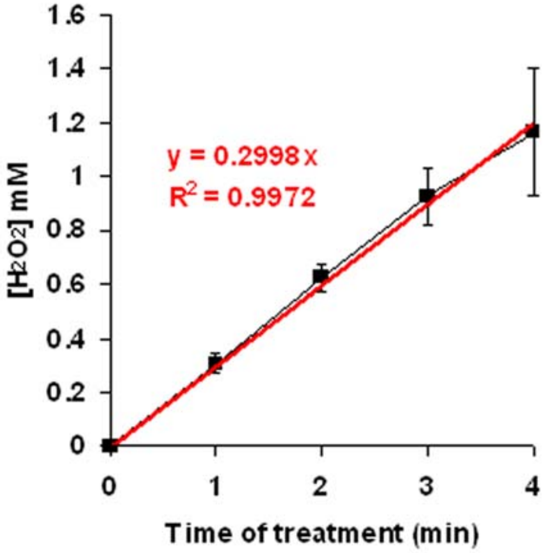

1.6
1.4
1.2
1
0.8
0.6
0.4
0.2
0
[H_2O_2] mM
y = 0.2998x
R^2 = 0.9972
0
1
2
3
4
Time of treatment (min)

Figure S3

A

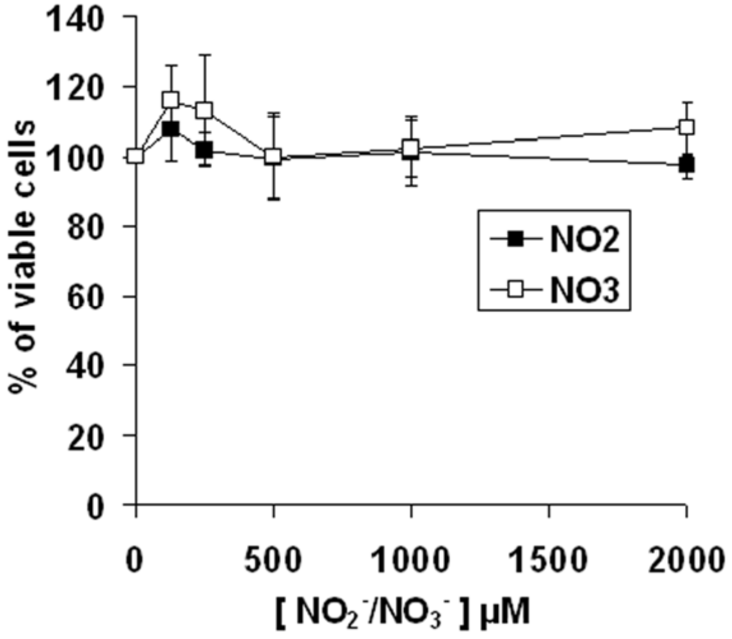

140
120
100
80
60
40
20
0
% of viable cells
0
500
1000
1500
2000
[NO_2^-/NO_3^-] µM
NO2
NO3

B

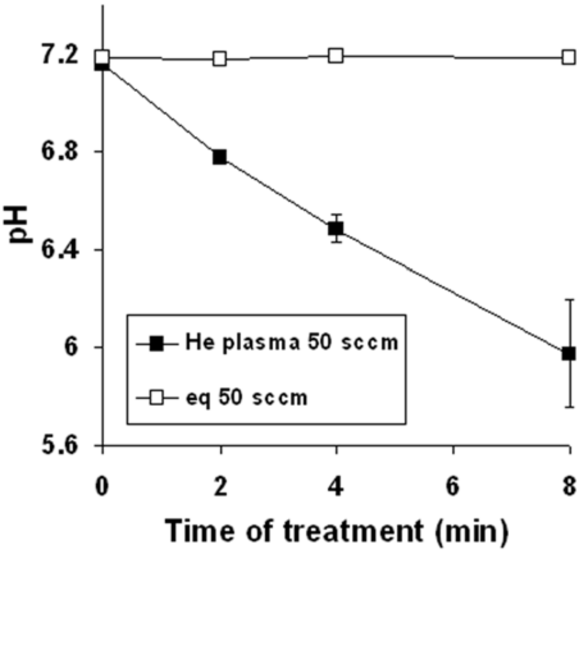

7.2
6.8
6.4
6
5.6
pH
0
2
4
6
8
Time of treatment (min)
He plasma 50 sccm
eq 50 sccm

Figure S4

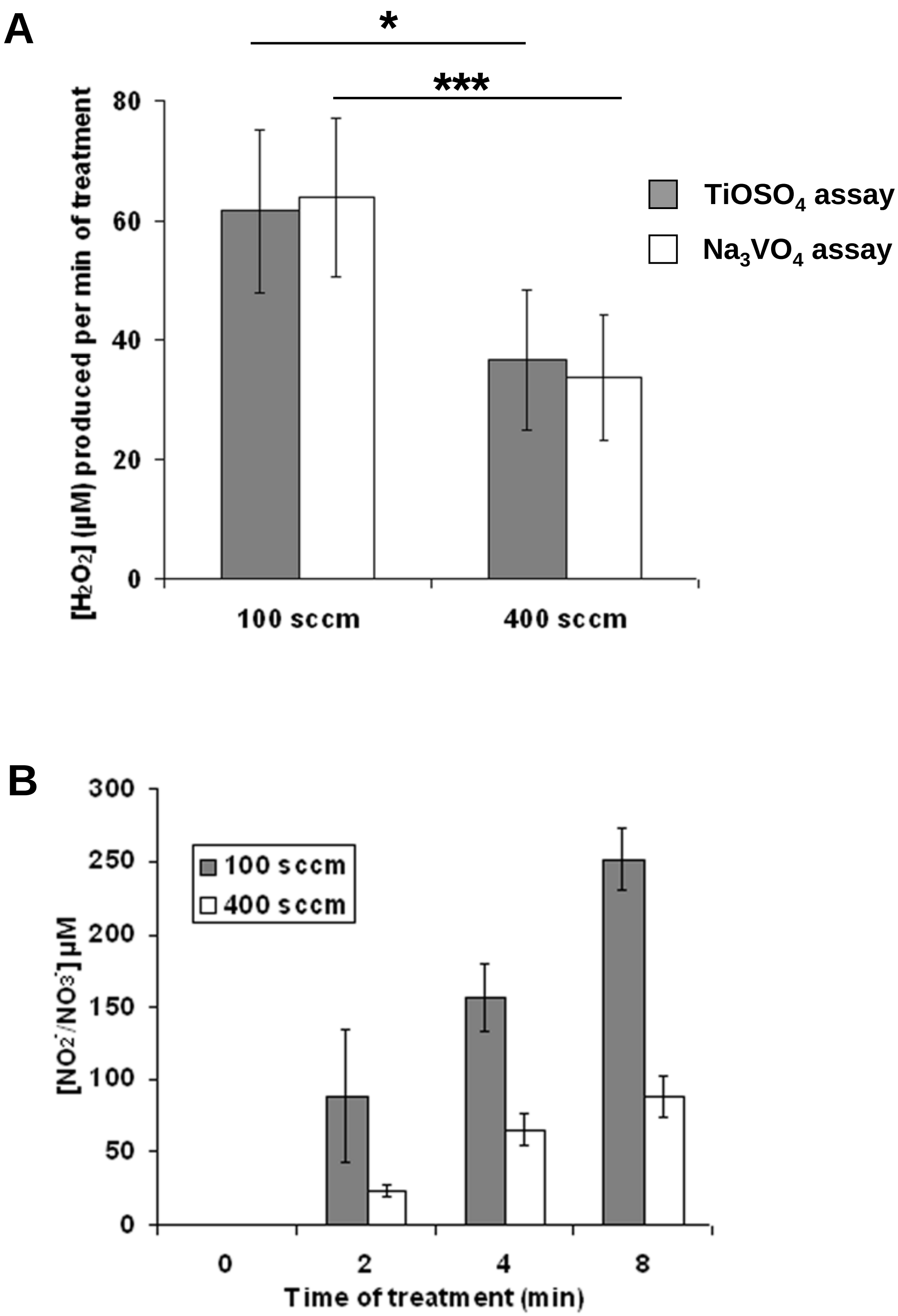
A
*
***
[H2O2] (µM) produced per min of treatment
80
60
40
20
0
100 sccm
400 sccm
TiOSO4 assay
Na3VO4 assay
B
300
250
200
150
100
50
0
[NO2-/NO3-] µM
100 sccm
400 sccm
0
2
4
8
Time of treatment (min)

Figure S5

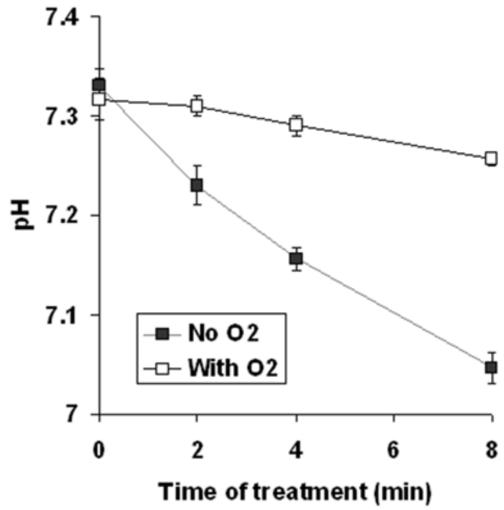

Figure S6

Supplementary Figure Legend

Figure S1. (**A**) Photograph of the home-made system used to shield the He flow from ambient air. (**B**) Schematic representation of the system. The external diameter of the needle is 1.6mm; the internal diameter of the needle is 1.10mm; the external diameter of the quartz tube is 4.30mm; the internal diameter of the quartz tube is 1.60mm; and the external diameter of the shielding tube is 32mm. The distance between the grounded electrode and the ending of the quartz tube is 5 mm.

Figure S2. Optical densities in the UV range (from 200 nm to 400 nm) of increasing concentrations of Na_3VO_4 diluted in PBS(Ca^{2+}/Mg^{2+}). The spectra were recorded on a double beam spectrophotometer (UVIKON XS, SECOMAM®, Servilab, France).

Figure S3. (**A**) Increasing concentrations of H_2O_2 were added to solutions of 2% $TiOSO_4$ diluted in 3 M H_2SO_4. The absorbance of the solutions at 407 nm was recorded and plotted as a function of the concentration of H_2O_2. The data are the mean ± SD of 5 independent experiments. (**B**) Five hundred microliters of PBS(Ca^{2+}/Mg^{2+}) were exposed for the indicated periods of time to He plasma at a gas flow of 50 sccm. The treated PBS(Ca^{2+}/Mg^{2+}) solutions were mixed to $TiOSO_4$ solutions and the absorbances recorded at 407 nm. The concentration of H_2O_2 in each condition was then determined using the equation shown in Figure S3A and plotted as a function of treatment time. The data are the mean ± SD of 6 independent experiments.

Figure S4. (**A**) MRC5Vi cells were seeded in 12 well plates and exposed for 1 h to increasing concentration of NO_2^- or NO_3^- diluted in 500 µl of PBS(Ca^{2+}/Mg^{2+}). Thereafter, 2.5 ml of

complete medium containing serum were added and the cells incubated in a humidified atmosphere at 37°C / 5% CO_2. Twenty-four hours later, the cell viability was assessed by a MTT assay. The data are the mean ± SD of 6 and 5 independent experiments for NO_2^- and NO_3^-, respectively. (**B**) Five-hundred microliters of PBS(Ca^{2+}/Mg^{2+}) were set per well in a 12 well plate and exposed to He plasma at a gas flow of 50 sccm for the indicated periods of time. In parallel, 500 µl of PBS(Ca^{2+}/Mg^{2+}) containing the equivalent expected concentrations of H_2O_2/NO_2^-/NO_3^- (for each time point) were also set per well. Thereafter, the pH of each solution was taken. The data are the mean ± SD of 3 to 4 independent experiments. For the reconstituted media (labelled "H_2O_2/NO_2^-/NO_3^- eq 50 sccm"), the error bars lie within the size of the symbols.

Figure S5. (**A**) The concentration of H_2O_2 produced per minute in 3 ml of PBS(Ca^{2+}/Mg^{2+}) by a He plasma jet at 100 and 400 sccm (conditions without a shielding gas of oxygen) and at an output voltage of 5.5 kV was determined by the Na_3VO_4 and $TiOSO_4$ assays. The data are the mean ± SD of 5 independent experiments for all the assays. Student t-test was used to check the statistical significance (*$p<0.05$, **$p<0.01$, ***$p<0.001$). (**B**) In parallel, the concentrations of NOx (NO_2^- + NO_3^-) were determined using the Griess assay. The data are the mean ± SD of 2 independent experiments.

Figure S6. Three milliliters of PBS(Ca^{2+}/Mg^{2+}) were set per well in a 12 well plate and exposed to He plasma at a gas flow of 100 sccm in the presence (with O_2) or absence (no O_2) of a shielding gas of oxygen for the indicated period of times, and at a output voltage of 5.5 kV. The pH of plasma-treated solutions was measured immediately after the treatment. The data are the mean ± SD of 3 independent experiments.
